# Pediatric hemophagocytic lymphohistiocytosis with predominant CNS involvement: MRI features and differential diagnosis

**DOI:** 10.1186/s12887-026-06829-z

**Published:** 2026-04-16

**Authors:** Osman Safa Bulut, Turgut Seber, Mehmet Akif Dündar, Fatoş Tekelioğlu

**Affiliations:** 1 Department of Radiology, University of Health Sciences, Kayseri City Training and Research Hospital, Kayseri, Turkey; 2 Department of Pediatrics, University of Health Sciences, Kayseri City Training and Research Hospital, Kayseri, Turkey; 3 Department of Pathology, University of Health Sciences, Kayseri City Training and Research Hospital, Kayseri, Turkey

**Keywords:** Hemophagocytic lymphohistiocytosis, Central nervous system, Pediatric intensive care, Histiocytic disorders, Hemophagocytosis

## Abstract

**Background:**

Hemophagocytic lymphohistiocytosis (HLH) is a rare, life-threatening hyperinflammatory disorder resulting from immune dysregulation that leads to uncontrolled macrophage activation. The diagnosis of HLH is often challenging, as its clinical manifestations can mimic various systemic conditions, including sepsis, autoimmune disorders, and malignancies. This overlap, combined with the rapid progression of the disease, may delay treatment and increase morbidity and mortality. Neurological involvement is particularly frequent in pediatric cases. Neuroimaging plays a critical role in raising suspicion for HLH, especially when classical clinical criteria are incomplete.

**Case presentation:**

We report a 3-year-old male patient who presented with growth retardation and long-standing polyuria and polydipsia, followed by acute respiratory distress requiring intensive care admission. During hospitalization, neurological deterioration prompted cranial magnetic resonance imaging (MRI). Imaging revealed diffuse infiltrative lesions involving the cerebral cortex, deep gray matter, white matter, brainstem, corpus callosum, and optic chiasm, with marked perivascular and leptomeningeal contrast enhancement, microhemorrhages, and cerebral venous thrombosis. Due to progressive neurological involvement and atypical imaging findings, a brain biopsy was performed, demonstrating histiocytic infiltration and hemophagocytosis. According to the HLH-2004 diagnostic criteria, the overall clinical, laboratory, and histopathological findings were consistent with hemophagocytic lymphohistiocytosis (HLH). Treatment was initiated according to the HLH-2004 protocol; however, the disease followed an aggressive course with a fatal outcome.

**Conclusion:**

This case highlights the diagnostic value of neuroimaging in pediatric HLH with predominant CNS involvement and underscores the importance of considering HLH in the differential diagnosis of diffuse infiltrative brain lesions. Brain MRI findings, particularly when correlated with histopathology, can play a crucial role in establishing the diagnosis and guiding timely management.

## Background

Hemophagocytic lymphohistiocytosis (HLH) is a rapidly progressive, potentially life-threatening hyperinflammatory syndrome that results from pathological activation of the immune system. The clinical picture is characterized by uncontrolled cytokine release (cytokine storm) and multiorgan dysfunction, primarily due to excessive activation of cytotoxic T lymphocytes and macrophages [1]. HLH is etiologically classified into two main groups: primary (familial) and secondary (acquired). Primary HLH typically presents in childhood and is associated with mutations in genes that regulate immune function (such as in Chédiak-Higashi syndrome 1 and Griscelli syndrome 2). Secondary HLH can occur at any age and develops in response to triggers such as infections (particularly herpesviruses like Epstein-Barr virus), malignancies (especially hematologic neoplasms), or autoimmune diseases [1–3].

Diagnosing HLH is highly challenging, as its clinical features often resemble those of sepsis, autoimmune diseases, or malignancies. Patients may present with nonspecific findings such as prolonged fever, splenomegaly, cytopenias, liver dysfunction, and neurological symptoms [2]. The rarity of HLH, variability of clinical manifestations, and lack of specificity in laboratory data further complicate the diagnostic process. Combined with the rapid progression of the disease, this can result in delayed treatment and increased morbidity and mortality [2, 3]. In this case report, we aim to present a 3-year-old patient who presented with growth retardation and diabetes insipidus and was diagnosed with HLH through histopathological examination. We focus on the neuroradiological imaging findings to highlight the role of imaging in the diagnostic process.

## Case presentation

**Fig. 1:**
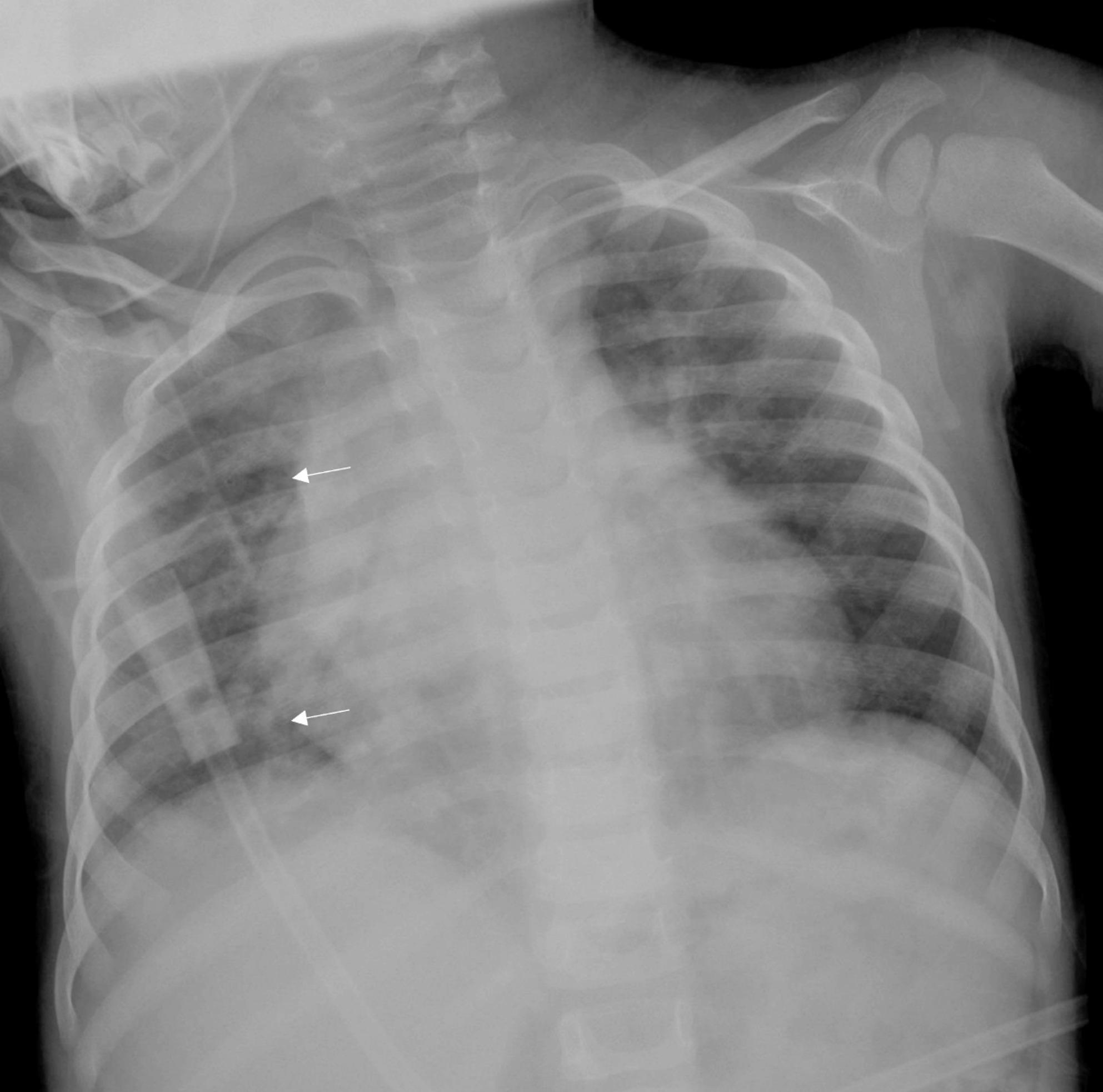
Chest X-ray demonstrating diffuse patchy pulmonary infiltrates (arrows)

A 3-year-8-month-old boy who had been experiencing polyuria and polydipsia for approximately one year presented to our hospital two days prior with worsening productive cough, respiratory distress, and tachypnea. Initial chest radiography revealed pulmonary infiltrates (Fig. 1). As his respiratory condition deteriorated, he was intubated and admitted to the pediatric intensive care unit with a preliminary diagnosis of bronchopneumonia.

He was born at 31 weeks of gestation via cesarean section to consanguineous parents, with a birth weight of 1,160 g, and required two months of neonatal intensive care due to respiratory distress syndrome. At 3 years and 3 months of age, he underwent left mastoidectomy for acute otitis media complicated by grade 4 peripheral facial paralysis.

**Fig. 2:**
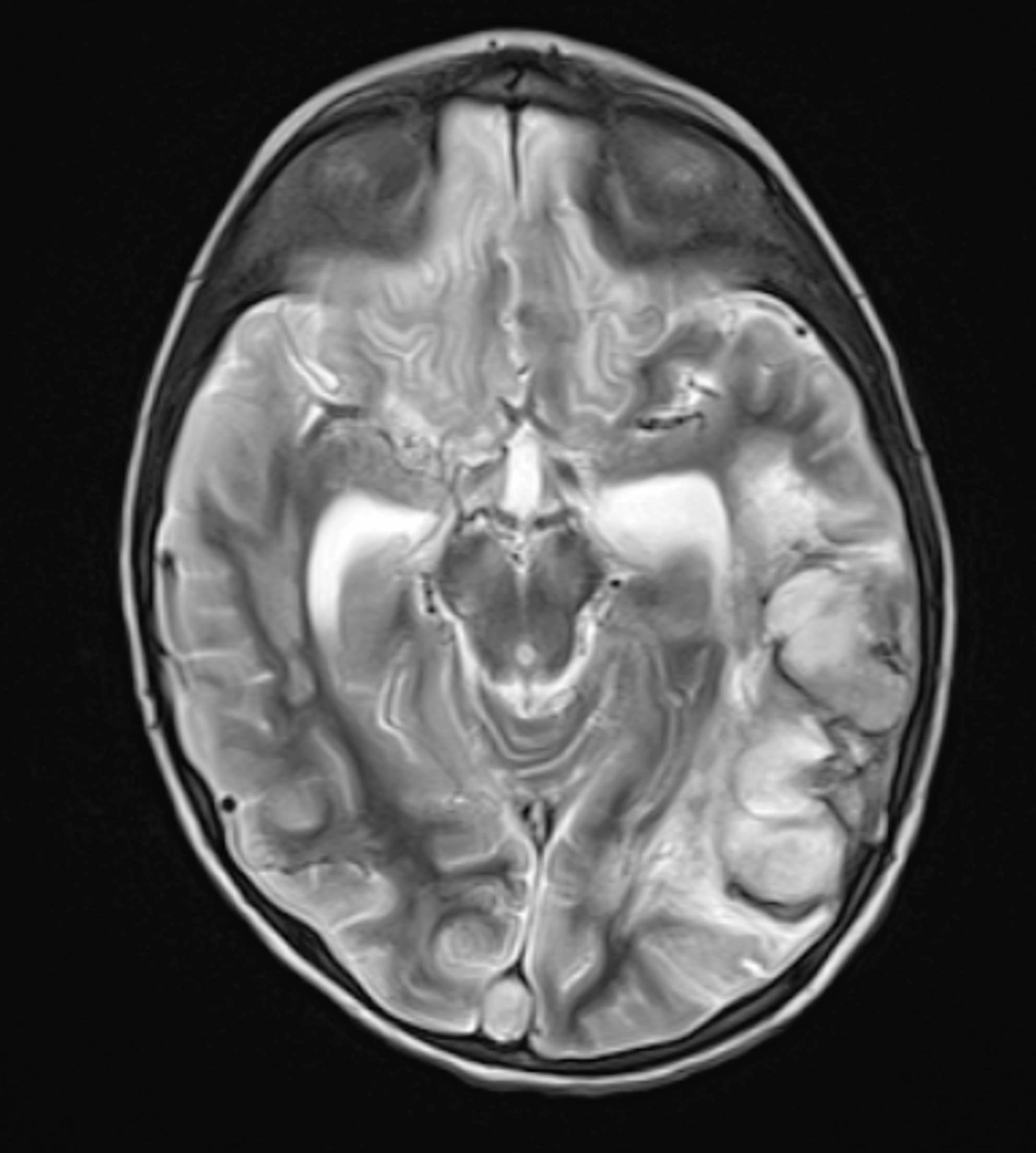
Axial T2-weighted image demonstrates diffuse hyperintense infiltrations involving the cerebral cortex, deep gray matter, white matter, and mesencephalon

**Fig. 3:**
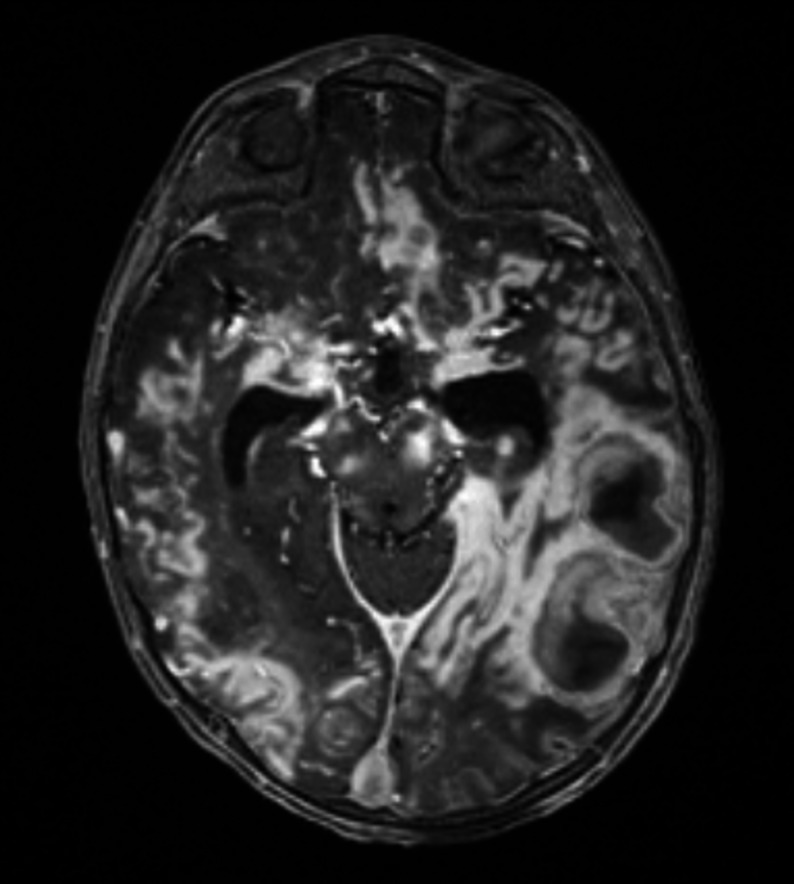
Post-contrast T1-weighted image (B) shows prominent bright enhancement of the cerebral parenchyma with characteristic perivascular and cortical “ribbon-like” involvement

**Fig. 4:**
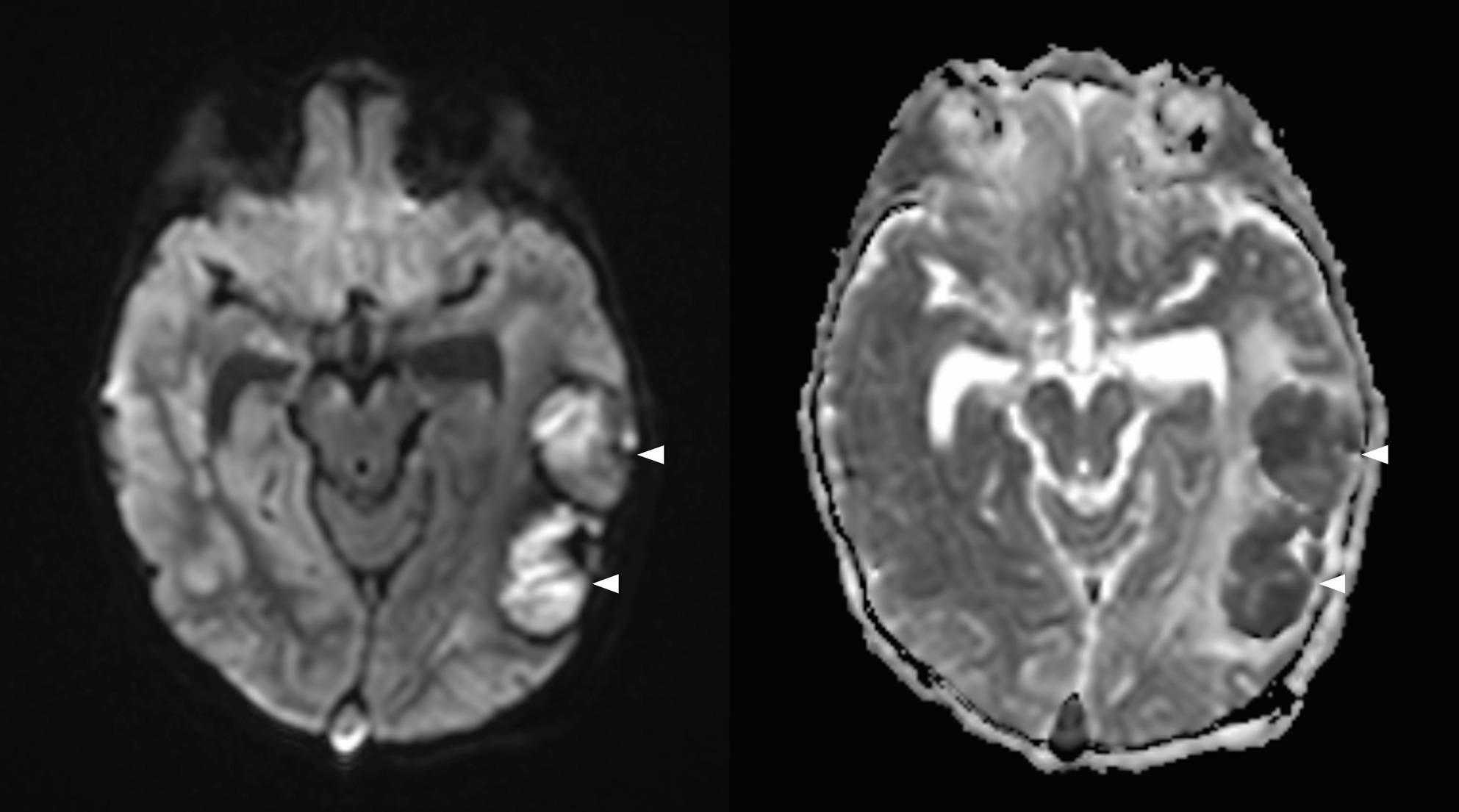
Diffusion-weighted image (DWI) and the corresponding ADC map reveal multiple areas of diffusion restriction consistent with parenchymal infiltration. Cytotoxic edema in the left posterior temporal region (arrowheads), likely representing acute Labbe venous thrombosis, is also present

**Fig. 5:**
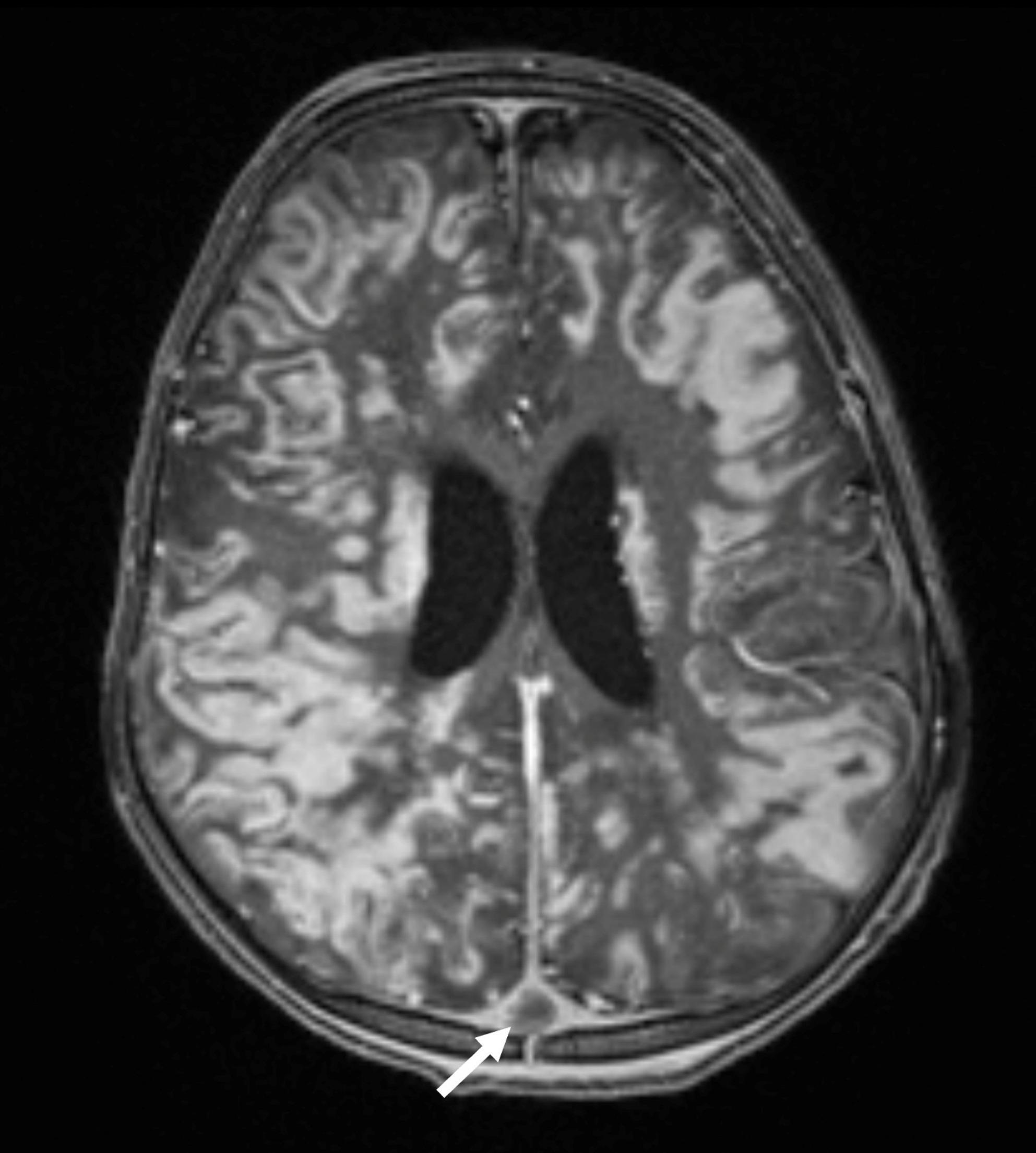
Contrast-enhanced T1-weighted image at the supratentorial level demonstrates diffuse enhancement in the cortical and subcortical regions, along with involvement of the corpus callosum. In addition, thrombosis is observed in the superior sagittal vein (arrow)

On the 13th day of hospitalization, the emergence of neurological symptoms including suspicious convulsive movements, loss of pupillary light reflex, anisocoria, and altered consciousness prompted cranial MRI. Imaging revealed diffuse and patchy contrast-enhancing infiltrates with restricted diffusion affecting the cerebral cortex, deep gray matter, white matter, midbrain, corpus callosum, and optic chiasm. Prominent bright parenchymal contrast enhancement displayed perivascular and cortical “ribbon-like” patterns. Thromboses were observed in several cerebral veins including superior sagittal, transverse, and sigmoid sinuses along with an acute venous infarct in the left posterior temporal region; microhemorrhages and vasogenic edema were noted in the thalamus, precuneus, and external capsule. The infundibulum appeared thickened due to infiltration, and the neurohypophysis showed loss of T1 hyperintensity. Bone marrow imaging revealed low T1 signal intensity consistent with an infiltrative process (Figs. 2, 3, 4 and 5).

Laboratory investigations revealed: serum ferritin 1,847 ng/mL (normal < 300 ng/mL), triglycerides 245 mg/dL (normal < 150 mg/dL), fibrinogen 187 mg/dL (low-normal, normal 200–400 mg/dL), hemoglobin 8.2 g/dL, platelets 89,000/µL, absolute neutrophil count 1,200/µL (pancytopenia), elevated LDH 856 U/L, AST 78 U/L, ALT 65 U/L, CRP 87 mg/L, ESR 45 mm/hr, and procalcitonin 2.3 ng/mL. Natural Killer (NK) cell activity and soluble IL-2 receptor levels were not available at our institution. The patient met 6 of 8 HLH-2004 diagnostic criteria: fever (which developed during hospitalization), pancytopenia, hypertriglyceridemia/hypofibrinogenemia, hyperferritinemia, splenomegaly and hemophagocytosis in bone marrow. Abdominal ultrasonography revealed hepatomegaly and mild splenomegaly.

Notably, the patient had a documented history of central diabetes insipidus diagnosed approximately one year prior to current admission, treated with desmopressin, along with growth failure (height z-score − 3.2 SD, weight z-score − 3.5 SD) and hypothyroidism on levothyroxine replacement. These chronic features suggested pre-existing hypothalamic-pituitary infiltration, likely representing an underlying chronic CNS histiocytic process that remained relatively quiescent prior to the acute presentation. The differential diagnosis included chronic histiocytic disorders such as Langerhans cell histiocytosis or Erdheim-Chester disease with subsequent secondary hemophagocytic activation triggered by respiratory infection. Following pediatric hematology consultation, further investigations revealed suspicious histiocytic cells on peripheral blood smear and definitive hemophagocytic cells were clearly identified in bone marrow biopsy.

**Fig. 6:**
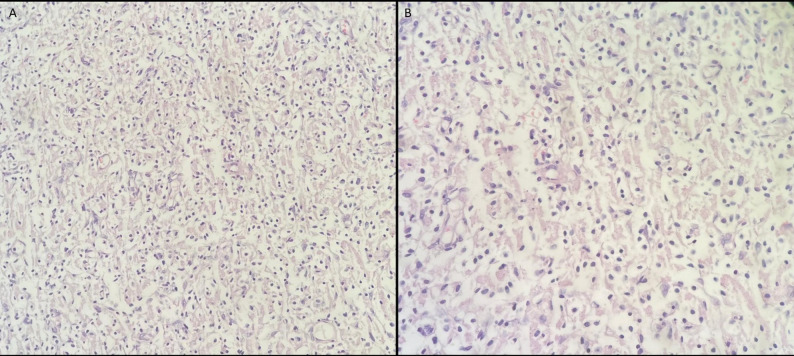
Hematoxylin and eosin–stained sections of a biopsy specimen obtained from the superficial cerebral cortex, showing diffuse infiltration of histiocytic cells with irregular cytoplasmic features (A, ×100) and a higher magnification view (B, ×200)

**Fig. 7:**
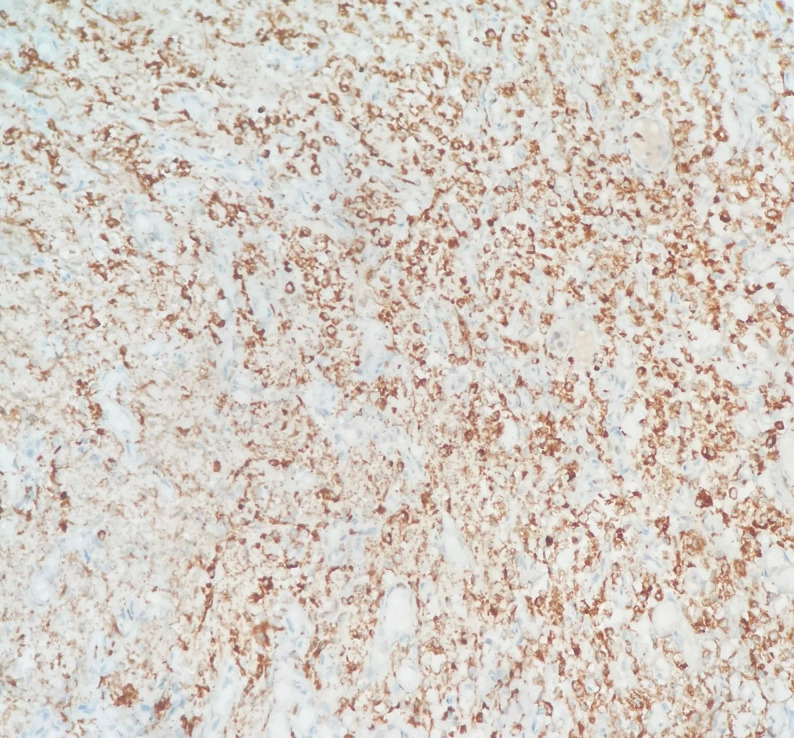
Diffuse cytoplasmic positivity of histiocytic cells for the CD68 immunohistochemical marker in the biopsy specimen from the superficial cerebral cortex (×200), confirming the histiocytic nature of the infiltrating cells

Brain biopsy from the superficial cortex demonstrated widespread histiocytic infiltration with hemophagocytosis (Fig. 6). Hematoxylin and eosin staining showed diffuse involvement of the tissue architecture by these cells. Immunohistochemistry revealed diffuse cytoplasmic CD68 positivity, confirming their histiocytic nature, while S100, CD1a, and Langerin were negative, making Langerhans cell histiocytosis less likely (Fig. 7). The proliferative index was low (Ki-67: 1–2%).

A multidisciplinary council on the 29th day of hospitalization, involving pediatric intensive care, hematology, pathology, radiology, and infectious disease specialists, established the working diagnosis of CNS-predominant HLH (or hemophagocytic syndrome in the setting of chronic histiocytic disorder) based on the combination of radiological findings, clear demonstration of hemophagocytic cells in bone marrow, fulfillment of 6/8 HLH-2004 criteria, and clinical presentation. Genetic testing for familial HLH–associated genes (PRF1, UNC13D, STX11, STXBP2, RAB27A) was negative; therefore, the patient was considered to have secondary HLH.

Treatment was initiated according to the HLH-2004 protocol, including etoposide, dexamethasone, intrathecal methotrexate, and cyclosporine A. The patient showed initial clinical improvement with successful extubation on day 41, supporting the therapeutic approach. Despite intensive treatment, the patient succumbed to the disease on the 90th day of hospitalization.

## Discussion

Hemophagocytic lymphohistiocytosis (HLH) is an aggressive and potentially fatal hyperinflammatory syndrome that arises due to dysregulation of the immune system. Dysfunction of cytotoxic T lymphocytes and NK cells leads to uncontrolled activation of macrophages. These macrophages infiltrate tissues and produce excessive levels of cytokines, resulting in multiple organ dysfunction [4]. This pathophysiological process accounts for the multisystemic involvement and highly variable clinical manifestations of HLH [2].

Patients with HLH typically present with severe systemic symptoms, and the clinical picture often mimics sepsis. The most common findings include prolonged fever, pancytopenia, and hepatosplenomegaly. Neurological involvement is frequent, particularly in pediatric cases, and central nervous system (CNS) manifestations are present in approximately 30–50% of patients at the time of diagnosis. These manifestations may include headache, encephalopathy, seizures, altered consciousness, cranial nerve palsies, and even coma [2].

Pulmonary findings may include dyspnea, cough, bilateral infiltrates, and pleural effusion, which can mimic acute respiratory distress syndrome. Hepatic involvement commonly presents with hepatomegaly, elevated transaminases, hyperbilirubinemia, coagulation abnormalities, hypofibrinogenemia, and disseminated intravascular coagulation (DIC). Additionally, lymphadenopathy and cutaneous findings such as petechiae, purpura, and erythema may be observed. As the disease progresses, systemic complications including hypotension and renal failure may also develop [2].

Diagnosis is based on the HLH-2004 guidelines, and one of the following two conditions must be met [5]:

- A molecular diagnosis consistent with HLH or
- The presence of at least five of the following eight criteria:
  1. Prolonged fever
  2. Splenomegaly
  3. Cytopenia affecting at least two cell lineages in the peripheral blood
  4. Hypertriglyceridemia and/or hypofibrinogenemia
  5. Hemophagocytosis in bone marrow, spleen, or lymph nodes
  6. Low or absent natural killer cell activity
  7. Hyperferritinemia
  8. Elevated levels of soluble interleukin-2 receptor (sCD25)

In HLH, brain MRI typically demonstrates widespread involvement of both gray and white matter, as well as leptomeningeal and perivascular enhancement, clearly reflecting histiocytic and lymphocytic infiltration [6, 7]. These lesions frequently exhibit diffusion restriction [7]. On SWI sequences, magnetic susceptibility artifacts due to microhemorrhages and calcifications are common [8]. MR spectroscopy often shows a decrease in N-acetylaspartate levels indicating neuronal necrosis along with elevated choline and lactate peaks. Additionally, hydrocephalus, cerebral venous sinus thrombosis, and optic nerve involvement have also been reported [6, 8].

In a retrospective cohort study conducted by Malik et al. in the pediatric population, two main MRI patterns were identified in children with HLH. Pattern 1, typically observed in older children (≥ 55 months), is characterized by an aggressive course and parenchymal lesions, presenting in three subtypes: multifocal white matter lesions, brainstem-dominant involvement, and cerebellitis. Pattern 2, seen in younger children (≤ 16 months), is associated with milder symptoms, higher survival rates without neurological deficits, and non-specific imaging findings [8].

When evaluating MRI findings, the differential diagnosis should include CLIPPERS (Chronic Lymphocytic Inflammation with Pontine Perivascular Enhancement Responsive to Steroids), Langerhans cell histiocytosis, Erdheim–Chester disease, Rosai–Dorfman–Destombes disease, CNS vasculitis, Glial Fibrillary Acidic Protein (GFAP) astrocytopathy, neurosarcoidosis, and intravascular lymphoma. The main differential diagnoses and their key MRI characteristics are summarized in Table 1.

**Table 1:** Imaging characteristics and steroid response in differential diagnosis of CNS hemophagocytic lymphohistiocytosis

Differential Diagnosis	CNS Localization & Distribution	Brain Imaging Features	Steroid Response
HLH (Hemophagocytic Lymphohistiocytosis)	Diffuse involvement of gray and white matter	Leptomeningeal and perivascular enhancement; diffusion restriction present; decreased NAA on MR spectroscopy	Steroid response often inadequate
CLIPPERS	Predominantly involves pons and cerebellum; may involve midbrain, medulla oblongata, and subcortical white matter	Perivascular linear enhancement; subtle T2 and FLAIR hyperintensities; no diffusion restriction; no mass effect; edema/hemorrhage/thrombosis rare	Generally good response to steroids
Langerhans cell histiocytosis	Predominantly involves the hypothalamic–pituitary axis (especially posterior pituitary)	Pituitary stalk thickening, loss of posterior pituitary bright spot on T1-weighted images	Steroid response often inadequate
Erdheim–Chester disease	Often involves meninges, hypothalamic–pituitary axis, brainstem, and cerebellum	Dural thickening or enhancing masses, pituitary stalk thickening, brainstem/cerebellar T2 hyperintense lesions. May mimic meningioma.	Steroid response often inadequate
Rosai–Dorfman–Destombes disease	Often involves dura and meninges. Usually focal mass-like lesions.	Extra-axial dural-based masses, homogeneous contrast enhancement. Mimics meningioma.	Steroid response often inadequate
CNS Vasculitis	Cortical and subcortical regions	Ischemic and hemorrhagic foci on MRI; vascular narrowing and beading on MR angiography	Generally good response to steroids
GFAP Astrocytopathy	Periventricular white matter	T2 hyperintensities in periventricular white matter; linear perivascular enhancement	Generally good response to steroids
Neurosarcoidosis	Perivascular areas extending from basal cisterns into brain parenchyma	Nodular perivascular enhancement; meningeal thickening	Good response to steroids in mild to moderate cases
Intravascular Lymphoma (IVL)	Small vessels	T2 hyperintense, dynamic ischemic lesions with contrast enhancement	Not applicable

CLIPPERS is predominantly observed in the pons and cerebellum, and less frequently in the midbrain, medulla oblongata, and cerebral white matter. On T2 and FLAIR sequences, it appears as subtle hyperintensities that become more pronounced after contrast administration, displaying perivascular linear enhancements resembling a “salt-and-pepper” pattern. Unlike HLH, the lesions are typically small and faint, with no mass effect. Diffusion restriction is usually less prominent than in HLH, and vasogenic edema, hemorrhage, or thrombosis are rare [9, 10]. In most CLIPPERS cases, contrast enhancement completely resolves following corticosteroid therapy, whereas in HLH and other non-CLIPPERS conditions, residual enhancement is more commonly observed [10].

Langerhans cell histiocytosis (LCH) is a clonal neoplastic proliferation of Langerhans cells most often involving the skeleton and skin, with up to ~ 80% of cases showing characteristic lytic bone lesions and frequent cutaneous involvement in infants and adults alike. Definitive diagnosis relies on histopathology demonstrating neoplastic Langerhans cells with grooved, coffee-bean nuclei and an inflammatory background, with immunohistochemistry positive for CD1a and/or langerin (CD207) as hallmark markers. LCH can also involve the hypothalamic–pituitary axis, typically manifesting on MRI as pituitary stalk thickening accompanied by loss of the normal posterior pituitary hyperintense signal on T1-weighted images. In multisystem LCH, corticosteroid therapy alone is generally insufficient and is typically administered in combination with chemotherapy. Vinblastine with prednisone is widely used as first-line treatment, whereas more intensive regimens may be required in patients with high-risk organ involvement. In contrast, single-system disease may be effectively managed with local therapies such as surgical excision or radiotherapy [11]. Unlike HLH, LCH does not present with the profound hyperferritinemia.

Erdheim–Chester disease is a rare clonal non-Langerhans cell histiocytosis that predominantly affects adults between 40 and 60 years of age, with pediatric cases being uncommon. It is characterized by multisystem infiltration of foamy histiocytes within a fibrotic stroma, frequently accompanied by Touton giant cells. Bilateral, symmetric osteosclerosis of the long bones of the lower extremities is a hallmark finding, present in most patients. Extra-osseous involvement commonly affects the retroperitoneum, cardiovascular system (e.g., “coated aorta”), lungs, orbits, and the hypothalamic–pituitary axis, often resulting in diabetes insipidus. Immunohistochemically, lesional histiocytes are CD68+, CD163+, and factor XIIIa+, but negative for CD1a and langerin. Activating MAPK pathway mutations, most commonly BRAF V600E, are identified in approximately half of cases. Treatment is guided by the underlying genetic alterations and disease extent. Patients with BRAF V600E mutations may benefit from BRAF inhibitors (vemurafenib and dabrafenib), while those with other MAPK pathway mutations can be treated with MEK inhibitors (cobimetinib and trametinib) [11].

Rosai–Dorfman–Destombes disease is a rare histiocytic disorder typically affecting young adults, characterized by large histiocytes showing emperipolesis of lymphocytes, plasma cells, or neutrophils. Nodal involvement is most common, particularly massive painless cervical lymphadenopathy, but extranodal sites including skin, bones, soft tissue, retro-orbital tissue, and CNS are frequently affected. Histologically, lesions consist of S100+, CD68+, and CD163 + histiocytes in a chronic inflammatory background; CD1a and langerin are negative. Treatment is guided by disease severity and extent. Mild or asymptomatic cases are usually observed, whereas localized lesions may be managed with surgical excision. Systemic therapy including corticosteroids, sirolimus, chemotherapy, immunomodulatory or targeted agents, and radiotherapy is reserved for unresectable or progressive disease [11].

CNS vasculitides are a group of disorders that typically occur with systemic inflammation and affect small- to medium-sized vessels. On MRI, bilateral cortical and subcortical ischemic and hemorrhagic foci at different stages are characteristic, while angiography commonly reveals vessel wall irregularities, narrowing, or a “beading” appearance [12]. The white matter involvement, marked perivascular infiltration, and contrast-enhancing meningeal lesions observed in HLH are atypical for vasculitis. Vasculitides generally respond well to corticosteroid therapy; however, in HLH, this response is often limited [12, 13].

Glial Fibrillary Acidic Protein (GFAP) Astrocytopathy is an immune-mediated inflammatory disorder of the central nervous system characterized by the presence of IgG antibodies against the GFAPα isoform in cerebrospinal fluid (CSF). The most typical finding is T2 hyperintensity and linear perivascular enhancement in the periventricular white matter [14]. The basal ganglia, thalamus, cortex, brainstem, hippocampus, and cerebellum may also be affected to a lesser extent. In general, patients show a good response to corticosteroid therapy [15].

Neurosarcoidosis is a rare granulomatous disease in the pediatric age group, usually identified in patients with systemic sarcoidosis [16]. It typically presents as nodular perivascular enhancement or plaque-like meningeal thickening extending from the basal cisterns into the brain parenchyma. Meningeal involvement may appear as either focal or diffuse thickening [17]. Treatment generally begins with corticosteroids, and mild-to-moderate cases often respond favorably to steroid therapy [18].

Intravascular lymphoma (IVL) is a lymphoproliferative disease characterized by the infiltration and proliferation of malignant B cells within the small blood vessel lumens, most commonly involving the central nervous system. It has been primarily described in adults and the elderly, while pediatric cases are extremely rare [19]. The disease presents with multifocal ischemic lesions [20]. The lesions demonstrate T2 hyperintensity and contrast enhancement. A dynamic disease course is typical of IVL, characterized by the regression of some lesions and the appearance of new ones over time. Microhemorrhages and signs of thrombosis may also be observed [19].

## Conclusion

Recognizing the pattern of central nervous system involvement in hemophagocytic lymphohistiocytosis (HLH) is crucial to prevent delays in diagnosis and treatment, especially in pediatric patients who do not meet the classic clinical diagnostic criteria. Clinically and radiologically, HLH can resemble many other diseases. In this study, the neuroimaging findings of HLH were discussed in detail and compared with several differential diagnoses. Therefore, adopting a multidisciplinary approach and carefully evaluating differential diagnoses are of great importance in the diagnostic process of HLH.

## Data Availability

All relevant data are included in this published article.

## Abbreviations

ADC: Apparent diffusion coefficient
CD68: Cluster of differentiation 68
CLIPPERS: Chronic lymphocytic inflammation with pontine perivascular enhancement responsive to steroids
CNS: Central nervous system
CSF: Cerebrospinal fluid
DI: Diabetes insipidus
DIC: Disseminated intravascular coagulation
DWI: Diffusion-weighted imaging
EBV: Epstein–Barr virus
FLAIR: Fluid-attenuated inversion recovery
GFAP: Glial fibrillary acidic protein
HLH: Hemophagocytic lymphohistiocytosis
IgG: Immunoglobulin G
IVL: Intravascular lymphoma
LCH: Langerhans cell histiocytosis
MRI: Magnetic resonance imaging
NAA: N-acetylaspartate
NICU: Neonatal intensive care unit
NK: Natural killer
sCD25: Soluble interleukin-2 receptor alpha
SWI: Susceptibility-weighted imaging
T1: T1-weighted imaging
